# Circulating Fibrocytes Are Increased in Neonates with Bronchopulmonary Dysplasia

**DOI:** 10.1371/journal.pone.0157181

**Published:** 2016-06-16

**Authors:** Chun Li, Xiaoyu Li, Chun Deng, Chunbao Guo

**Affiliations:** 1 Department of Pediatric General Surgery and Liver Transplantation, Children's hospital, Chongqing Medical University, Chongqing, 400014, P.R. China; 2 Department of Neonatology, Children’s Hospital of Chongqing Medical University, Chongqing, China; 3 Ministry of Education Key Laboratory of Child Development and Disorders, Children's hospital, Chongqing Medical University, Chongqing, 400014, P.R. China; University of Kansas Medical Center, UNITED STATES

## Abstract

**Background:**

Bronchopulmonary dysplasia (BPD) is characterized by the aberrant remodeling of the lung parenchyma, resulting from accumulation of fibroblasts or myofibroblasts. Circulating fibrocytes are implied in pulmonary fibrosis, but whether these cells are associated with the development of BPD or the progressive fibrosis is unknown. The aim of the present study was to investigate the occurrence of fibrocytes in peripheral venous blood and explore whether these cells might be associated with severity of BPD.

**Methods:**

We investigated circulating fibrocytes in 66 patients with BPD, 23 patients with acute respiratory distress syndrome(ARDS) and 11 normal subjects. Circulating fibrocytes were defined and quantified as cells positive for CD45 andcollagen-1 by flow cytometry. Furthermore, serum SDF-1/CXCL12 and TGF-β1 were evaluated using ELISA methods. We also investigated the clinical value of fibrocyte counts by comparison with standard clinical parameters.

**Results:**

The patients with BPD had significantly increased numbers of fibrocytes compared to the controls (p < 0.01). Patients with ARDS were not different from healthy control subjects. There was a correlation between the number of fibrocytes and pulmonary hypertension or oxygen saturation (p < 0.05). Fibrocyte numbers were not correlated with other clinical or functional variables or radiologic severity scores. The fibrocyte attractant chemokine CXCL12 increased in plasma (p < 0.05) and was detectable in the bronchoalveolar lavage fluid of 40% of the patients but not in controls.

**Conclusion:**

These findings indicate that circulating fibrocytes are increased in patients with BPD and may contribute to pulmonary fibrosis in BPD. Circulating fibrocytes, likely recruited through the CXCR4/CXCL12 axis, might contribute to the production of TGF-β1 for the expansion of fibroblast/myofibroblast population in BPD.

## Introduction

Today, BPD remains a serious challenge to the neonatologist, affecting approximately 25% of infants born weighing less than 1,500 g [1, 2]. Histopathologically, BPD was characterized by widespread interstitial fibrosis and impaired alveolarization, which is recognized as the prominent features of BPD [3, 4]. Fibroblasts, especially in their activated differentiated state, myofibroblasts, are considered to be key elements in the pathogenesis of fibrosis. A persistent increase in the number of fibroblasts is found both in the interstitium and in the airspaces of the lungs of infants with BPD [1]. Understanding the origin and recruitment of these cells, and the pathogenic mechanisms at play, should shed further insight into the basis for the development of progressive fibrosis and for the development of improved clinical management options for affected patients [5].

Circulating fibrocytes are the spindle-shaped progenitor cells, account for approximately 0.1–0.5% of peripherally circulating, non-erythrocyte cells, first described in 1994 as a distinct population of blood-borne cells, capable of elaborating various components of the extracellular matrix (ECM) and participate in many biological processes [6, 7, 8].Fibrocytes are identified by specific coexpress of several markers, such as mesenchymal markers including collagen-1, together with haematopoietic markers such as CD34, leukocyte markers such as CD45, as well as the fibroblast products α-smooth muscle actin (αSMA) and myeloid markers. Fibrocytes also express several chemokine receptors, particularly CXCR4, which has been shown to mediate the effect of stromal derived factor-1 (SDF-1/CXCL12), a specific ligand for CXCR4, in causing migration of fibrocytes in pulmonary fibrosis [9, 10]. The transforming growth factor-β1 stimulates the differentiation of fibrocytes into myofibroblast-like cells by stimulating the release of growth factors, supporting a potential role of these cells in myofibroblast differentiation [11].

In the context of normal wound healing and fibrotic progression, circulating fibrocytes may selectively migrate to injury sites, and differentiate in to fibroblast-like cells within areas of extracellular matrix deposition. Accumulating recent research indicated elevated circulating fibrocytes levels in human fibrotic interstitial lung disease [12, 13]. Furthermore, in vivo evidence in several models, supported the important role of fibrocytes in deposit ECM components during various fibroproliferative disorders of the lung, and inhibition of fibrocyte recruitment leads to a decrease in fibrosis [14]. Further, one report has implicated fibrocytes in human idiopathic pulmonary fibrosis (IPF) by showing elevated numbers in the blood of patients with IPF [15]. Fibrocytes have also been found in IPF lung tissue and the CXCR4-CXCL12 axis is likely implicated in their recruitment [16]. However, the presence of circulating fibrocytes during BPD pathogenesis remains undefined.

The purpose of this study was to test whether there could be an increased number of circulating fibrocytes in patients with BPD, and whether these increased fibrocyte levels may help to assist in early evaluation of BPD as well as monitoring for reparative process of disease. We therefore studied the number and activity of circulating fibrocytes in the peripheral blood of patients with BPD. We also quantified the plasma and bronchoalveolar lavage levels of TGF-b1 and SDF-1/CXCL12. Hereby we show that the number of fibrocytes identified in peripheral venous blood was elevated and correlated to the severity of BPD.

## Methods

### Patient population

The study protocol for consent, data collection, and privacy protection were approved by the Institutional Review Board of the Chongqing Medical University and performed in accordance with the ethical standards prescribed by the Helsinki declaration of the world medical association. The Children’s Hospital of Chongqing Medical University have the capacity of 1500 beds and could provide tertiary care in southwest China. NICU is a level III, 80-bed intensive care unit for the care of critically ill neonates and has 800–1000 admissions per year. A case-control study was performed from January 2008 to July 2015. Detailed clinical data from the medical records, including daily flow sheets, laboratory and radiographic reports, underlying illnesses, procedures, and medications were reviewed by one of the investigators. The patient records information was anonymized and de-identified prior to analysis. The involved demographic and perinatal characteristics were recorded, including gender, birth weight, gestational age, surfactant administration, pre-natal steroid administration, mode of delivery, and so on. Diagnosis of BPD was based on a modification of the National Institutes of Health workshop definition for infants born at gestational age < 32 weeks, i.e. treatment with supplemental oxygen for at least 28 d [17]. Criteria for enrollment included gestational age less than 32 weeks and birth weight between 500 and 1,250 g. Exclusion criteria were patients with presence of acute bacterial and the most common viral infections (including performing sputum or bronchoalveolar lavage fluid [BALF]analysis plus nasopharyngeal swab);patients with cardiac dysfunction (except patent ductus arteriosus [PDA], patent foramen ovale or atrial septal defect <1 cm, or ventricular septal defect <3 mm if known before enrollment) and ongoing infection. Additionally, to minimize severity differences in the study population, patients managed in the intensive care unit (ICU) for more than 45 days were excluded.

### Flow cytometric analysis

Thirty milliliters (3 ml) of peripheral venous blood samples were collected in sterile test tubes and frozen at -80°C until processing for flow cytometry (FCM) and enzyme-linked immunosorbent assay (ELISA) tested on BPD diagnosis and 5 and 9 months later. For FCM, 1 ml blood samples were incubated with ACK lysis buffer to remove red blood cells and then prepared to a concentration of 1.0×10^7^/ml in PBS containing 0.1% FBS. Following initial isolation, the mononuclear cells were first stained with the PerCP-conjugated surface antigen anti-CD45 (CD45–PerCP, BD Biosciences, San Jose, CA) or isotype control antibody (IgG1,BD Biosciences). The cells were then permeabilized with a Cytofix/Cytoperm kit (BD Biosciences) for intracellular antigens detection prior to intracellular staining ofcollagen-1 and αSMA. Next, the cell pellet was incubated with PE-conjugated anti-CD34 (BD Biosciences, San Jose, CA) and FITC conjugated anti–human collagen-1 antibody (Rockland Immunochemicals, Gilbertsville, PA) or IgG isotype control for 30 minutes. The cell suspension was analyzed with a BD FAC Scan flow cytometer (BD Biosciences, San Jose, CA) using Cellquest 3.2.1f1 software. All data were analyzed with FlowJo software (Tree Star, Inc., Ashland, OR). Baseline leukocyte counts were expressed as a percentage of total leukocyte counts.

### Measurement of SDF-1/CXCL12 and TGF-β1

The concentration of SDF-1/CXCL12, IL-1β and TGF-β1 in plasma and BAL samples was determined by ELISA using the commercially available enzyme-linked immunoabsorbent assays according to the manufacturer’s instructions (Quantikine, R&D Systems, Minneapolis, MN, USA). Optical densities were determined using a microtiter plate reader (Multiscan RC Type 351; Labsystems, Helsinki, Finland) at 405nm, with a correction wavelength set at 540 or 570 nm. The blank was subtracted from the duplicate readings for each standard and sample without any knowledge of survival or other clinical data. Concentrations are expressed as pg/mL for SDF-1/CXCL12 and ng/mL for TGF-β1 and IL-1β. All of the analyses and calibrations were carried out at least in duplicate. The mean values were used for statistical analyses.

### Statistical analysis

Statistical comparisons were carried out by GraphPad Prism 5.0 (GraphPad Software, La Jolla, CA) and SPSS 13.0 (SPSS Inc, Chicago, IL). Categorical and continuous variables were reported as frequencies (percentages) and mean ± SEM, respectively. For distributed continuous variables, the Student’s t-test and Mann-Whitney U test were used to compare normally and abnormally distributed variables. For discrete variables, chi-square test or Fisher’s exact test was selected. The Friedman’s test (when appropriate) was applied to compare temporal variations. The level of significance was adjusted for multiple comparisons by the Student-Newman-Keultest. Spearman's rank correlation coefficient Test were performed for the correlation analyses. Statistical significance was accepted for 2-sided P values of less than 0.05.

## Results

### Patient characteristics

At the time of the analysis, a total of 106 infants were enrolled between January 2008 and July 2015. Fifty patients were excluded from the study for the following reasons: ten patients with lethal congenital abnormality; and three patients for anticipated death before hospital discharge; eight patients lacked proper documentation, 19 patients had received steroids, and samples for 10 patients could not be appropriately collected or stored. Finally, 66 eligible and evaluable patients and 23 patients with acute respiratory distress syndrome(ARDS), and 11 age-matched healthy volunteers fulfilled the criteria for inclusion and enrolled in the study (Table 1).Among study cohorts, females accounted for 46.7%, and gender distribution (p>0.99) was similar among BPD, non-BPD patients and normal subjects. The baseline characteristics of infants at birth and of their mothers were similar in the 3 groups (Table 1).

**Table 1:** Comparison of demographic and clinical characteristics between infants with and without BPD.*

	BPD (n = 66)	ARDS(n = 23)	Normal Subjects(n = 11)
Birth weight (g)	1233 ± 167.2	1297 ± 158	1343 ± 136
Gestational age (wks)	29.3 (27–31)	30 (28–31)	30.5(29–32)
Female, *n* (%)	31 (46.7%)	11 (47.8%)	5 (45.5%)
Birth Head Circ (cm)	26.4 ± 5.2	26.7 ± 4.7	26.8 ± 4.2
Apgar score at 5 min	7.3 [6–9]	7.4 [7–9]	7.9 [6–10]
Admission age(days)	29±53	36±71	33±67
PDA, n (%)	27(40.9)	4(17.4)	0#
VSD, n (%)	3(4.5)	1(4.3)	1(9.1)
IVH, n (%)	7(10.6)	2(8.7)	1(9.1)
Vaginal delivery	24 (36.4%)	9(39.1%)	5 (45.5%)
Maternal smoking	7(10.6)	3 (13.0%)	2(18.2)
Post admission steroids	45(68.2)	7(30.4)	0#
Prenatal steroids	13 (92.8%)	45 (91.8%)	0#
Maternal pregnancy-induced hypertension	4 (28.5%)	17 (34.7%)	5(45.5)
Chorioamnionitis	4 (28.5%)	11 (22.4%)	2(18.2)
Cord blood pH	7.30 ± 0.07	7.29 ± 0.08	7.37± 0.06
Surfactant use	21 (31.8%)	9 (39.1%)	0#
Mechanical ventilation ≥ 1 week	26 (39.4%)	8 (34.8%)	0#
Sepsis	12 (18.2%)	3 (13.0%)	0#
Nitric Oxide, n(%)	11 (16.7%)	3 (13.0%)	0#
Steroids	48 (72.7%)	4(17.4)*	0#
Pneumothorax, n(%)	4(6.1)	1(4.3)	0
ROP, n (%)	8(12.1)	2(8.6)	0
Caffeine, n (%)	41 (62.1%)	13 (56.5%)	7(63.6)
Discharge weight (g)	3546±1152	3722±1523	4118±1484

* P<0.01, compared with patients with BPD.

# P<0.01 compared with patients with BPD.

### Fibrocytes in peripheral blood

**Fig 1:**
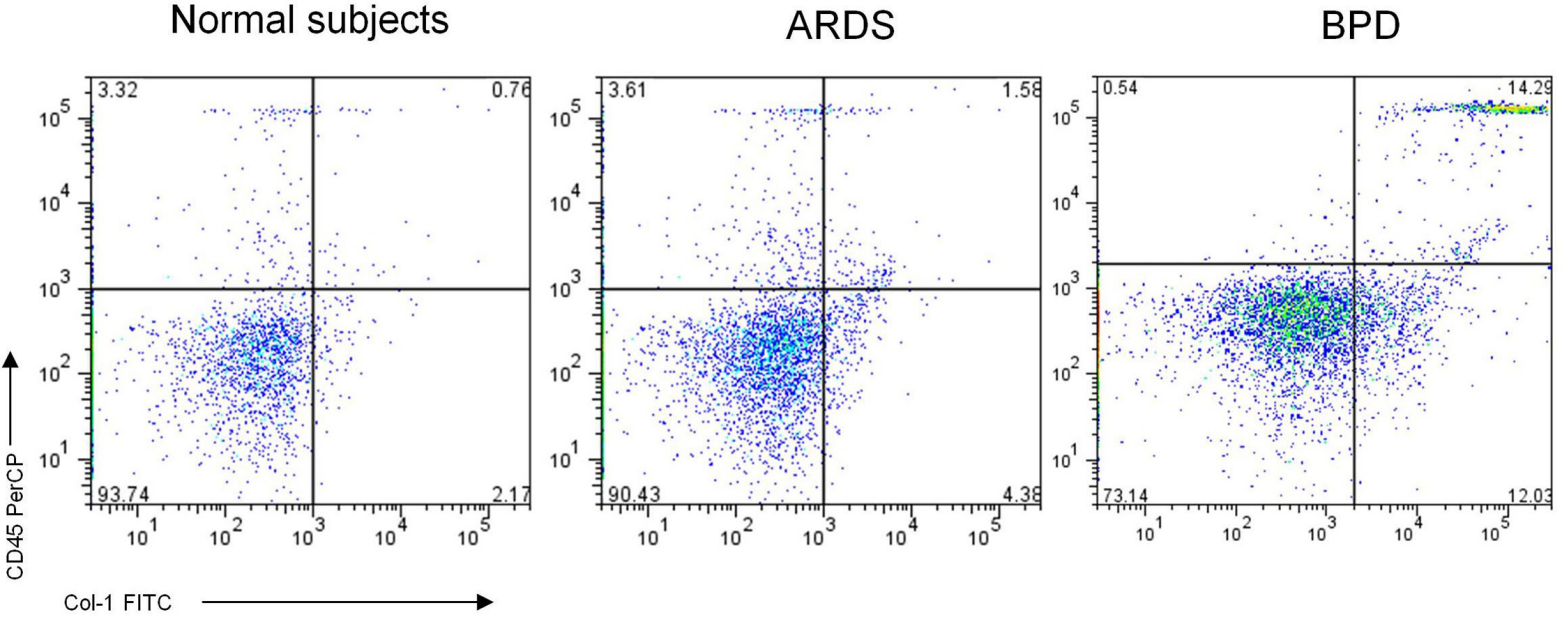
Flow cytometric analysis of Circulating fibrocytes on patients with BPD, ARDS and normal subjects by immunostaining of CD45, and collagen I (Col-I). FACS histogram illustrates representative of three independent experiments.

**Fig 2:**
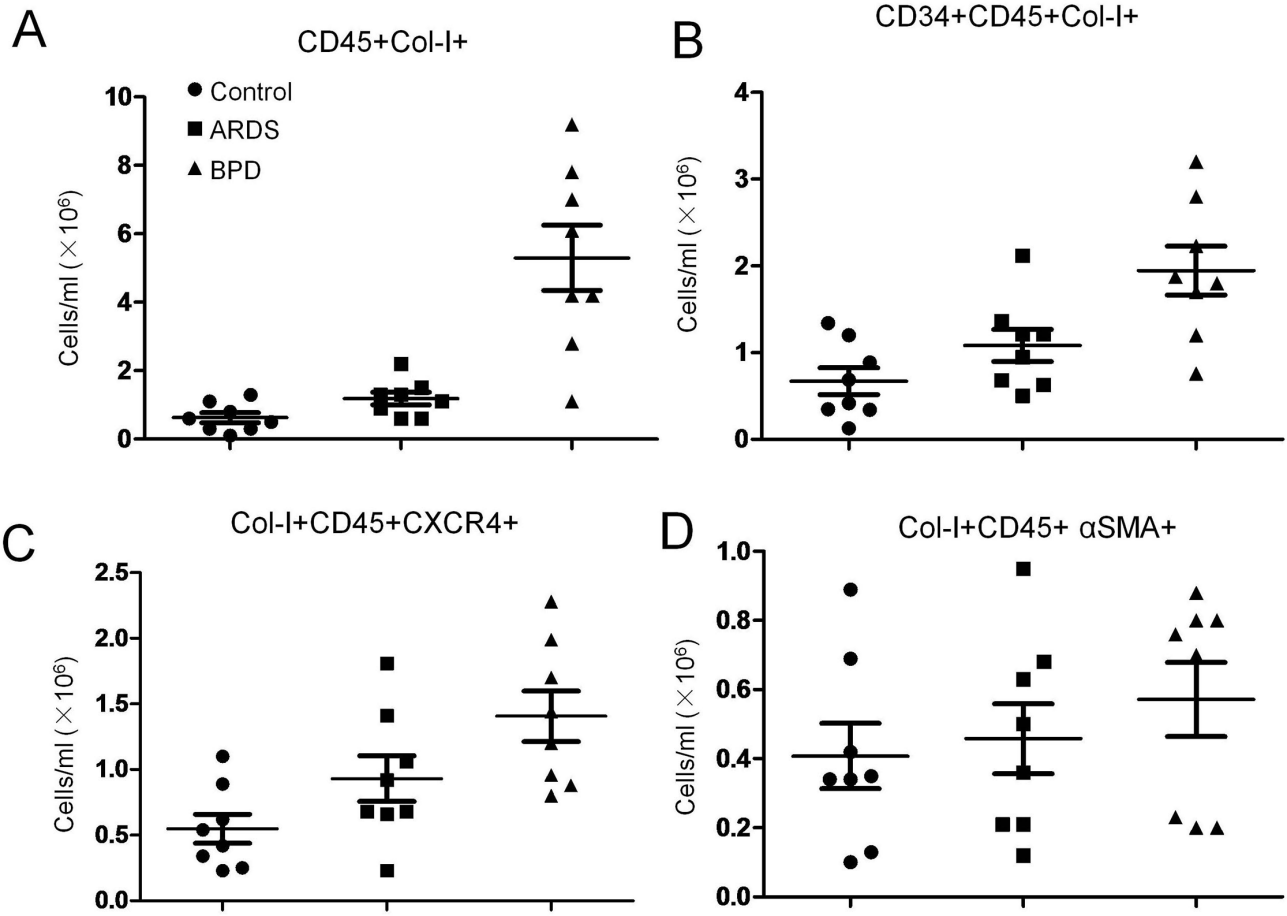
Flow cytometric analysis of circulating fibrocyte subpopulations on patients with BPD, ARDS and normal subjects. Scatter plot indicated average number of (A) CD45+Col-I+, (B) CD34+CD45+Col-I+, (C) Col-I+CD45+CXCR4+, (D) Col-I+CD45+ αSMA, were presented respectively. Horizontal lines represent mean ± SEM.

**Fig 3:**
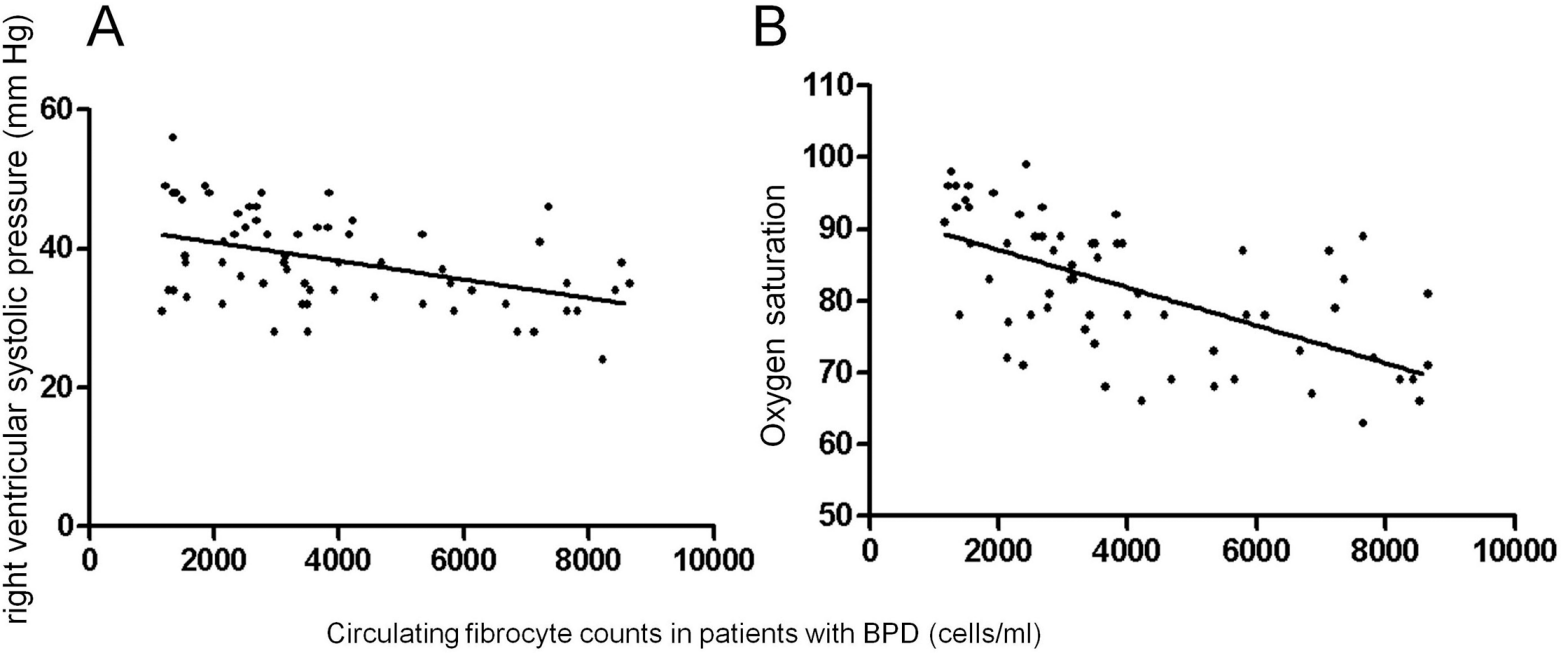
Correlation between circulating fibrocyte counts and right ventricular systolic pressure (A) (Pearson correlation coefficient; r = −0.62; p < 0.01) or oxygen saturation (B) (Pearson correlation coefficient; r = −0.62; p < 0.01).

Using quantitative flow cytometry analysis (Fig 1), overall circulating fibrocyte counts, defined as CD45and collagen-1expressing (CD45+Col-1+) cells in the peripheral blood was up to 5-fold higher in patients with BPD compared with in patients with ARDS (p < 0.01) and normal subjects (p < 0.01) (Fig 2A). The plasma circulating fibrocyte counts positively correlated with pulmonary hypertension (PH)(r = −0.46; p < 0.05) and oxygen saturation (Pearson correlation coefficient; r = −0.62; p < 0.01) (Fig 3A and 3B) but not with any other clinical or functional variables including disease severity and bronchoalveolar lavage inflammatory factors.

**Fig 4:**
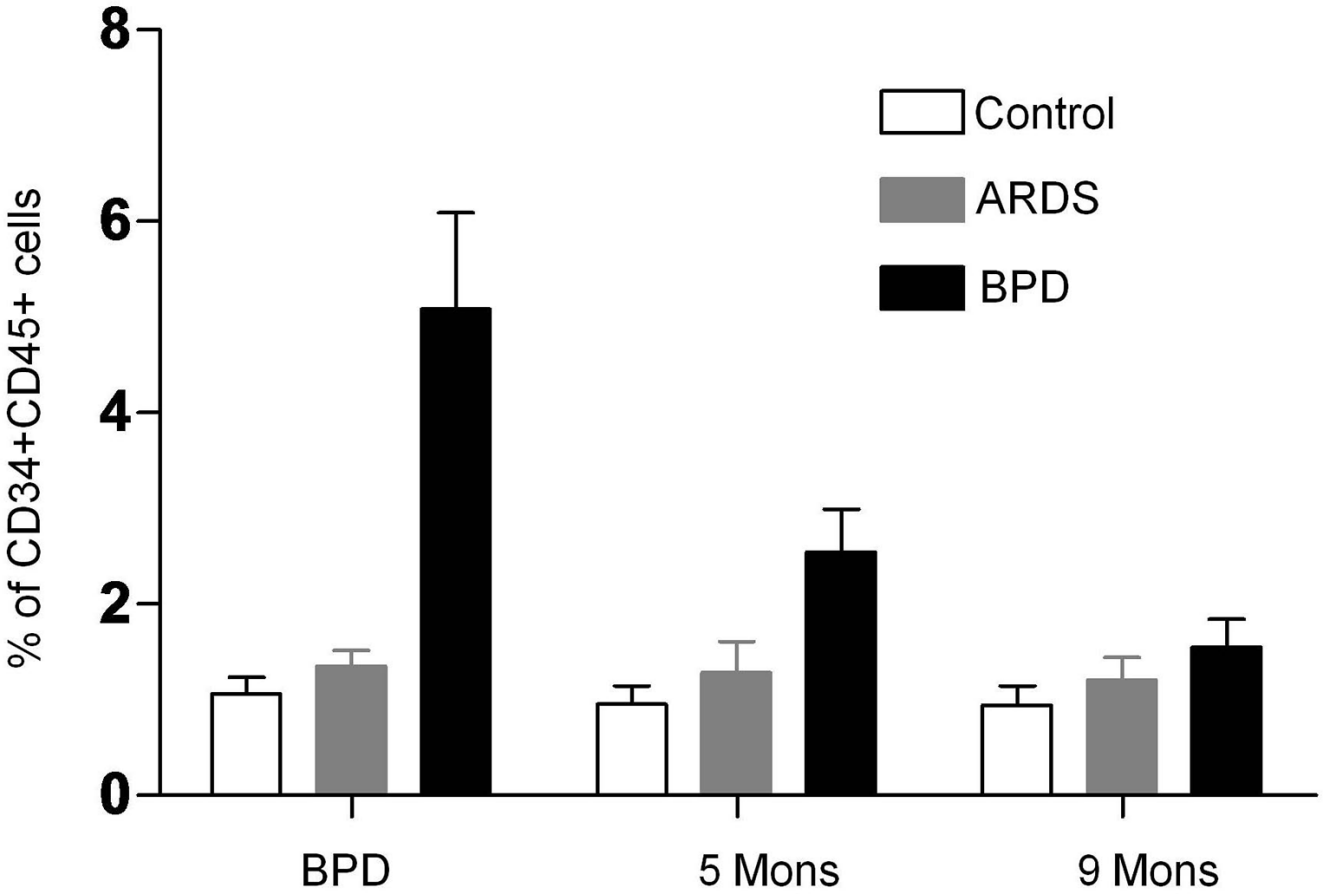
Flow cytometric analysis of circulating fibrocyte on patients with BPD, ARDS and normal subjects on different time points. Histogram indicated average values of a minimum of three independent experiments; bars: ± SEM. n = 3 per group. *P < 0.05, #P < 0.05, one-way ANOVA.

In patients with BPD, circulating fibrocyte was markedly elevated and maintaining high levels on 5 months later, returned to nearly baseline values on9months later. Fibrocyte counts in patients with ARDS were not different from those of control subjects (Fig 4). Similarly, when expressed as absolute counts per milliliter of blood, fibrocytes were also increased in patients with BPD compared with in normal subjects. Similar trends were observed for circulating levels of bone marrow-derived fibrocytes (CD34+CD45+Col-I+ cells) in the peripheral blood, with higher in patients with BPD compared with in normal subjects (Fig 2B). However, these differences failed to reach statistical significance.

To further characterize circulating fibrocytes, we analyzed freshly isolated peripheral blood mononuclear cells by triple-staining for Col-I, CD45, andCXCR4 or αSMA by flow cytometry. We found that the majority of CD45+Col-I+ cells expressed CXCR4 (Fig 2C), and all measured counts of differentiated fibrocyte populations CD45+Col-I+ αSMA+cells (Fig 2D) were higher among patients with BPD compared to normal control.

### Plasma and BAL levels of SDF-1/CXCL12 and TGF-β1 are increased in BPD patients

Derived from bone marrow, fibrocytes (CD45+Col1+CXCR4+) are traffic to injured tissues in response to numerous microenvironmental cytokines, like the CXCR4/CXCL12 chemokine axis. To assess whether increased SDF-1/CXCL12 was accompanied by the fibrocytes trafficing during BPD, we measured the concentration of SDF-1/CXCL12, the ligand of CXCR4 in plasma and bronchoalveolar lavage from patients with BPD, ARDS and normal subjects.

**Fig 5:**
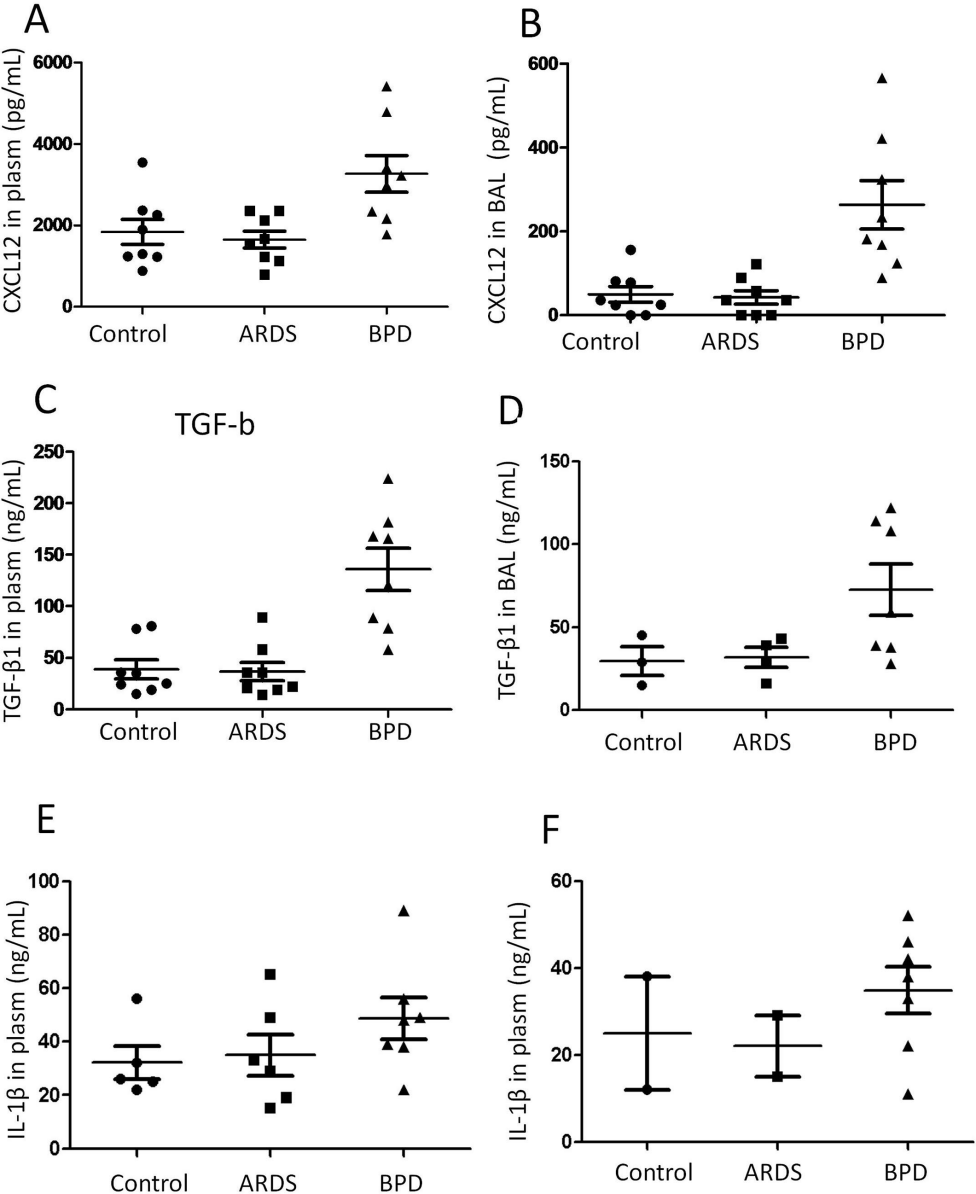
Comparison of SDF-1/CXCL12, TGF-b1, IL-1b from plasm (A) and BLD(B) as indicated within the figures on patients with BPD, ARDS and normal subjects.

As illustrated in Fig 5, the concentration of SDF-1/CXCL12was significantly increased in patients with BPD compared with patients with ARDS and normal subjects. In the patients with ARDS, there was also a significant increase in serum SDF-1/CXCL12 compared with those in normal subjects (P<0.01, two-way ANOVA; Fig 1). This suggests a correlation between serum SDF-1/CXCL12 and pulmonary diseases. In patients with BPD, the serum TGF-β1 and IL-1β was also markedly rose, compared with patients with ARDS and in normal subjects (Fig 5A). SDF-1/CXCL12 was detectable in the BAL fluid of 85%of patients with BPD and in 11% of the normal controls (Fig 5B). At the same time, TGF-β1 and IL-1β were significant increase in BAL fluid for all the patients, whereas they were almost absent in normal subjects.

## Discussion

This study shows an increased number of circulating fibrocytes (CD45+Col-I+) in the peripheral blood of patients with BPD, but not in normal subjects. During disease recovery, fibrocyte counts decreased to an average of 1.0% of peripheral blood leukocytes in 5 months and fell back to normal levels in 9 months. We also quantified elevated circulating fibrocytes in patients with ARDS, assuming that their acutely injured lungs are active in creating the repair signals for circulating fibrocytes recruietment. Similar differences were detected for other circulating fibrocyte subpopulations, including CD45+Col-1+ CDCR4+cells, furthermore, BAL fluid SDF-1/CXCL12 level was elevated in patients with BPD, implying the recruiting capablity of these extrapulmonary cells. At the same time, the enhanced TGF-β1 in BAL fluid, impling a proliferative and differentiation capacity of circulating fibrocytes, support a potential role for these circulating progenitor cells in the persistence and progression of interstitial fibrosis in BPD.

Although incompletely understood, circulating fibrocytes are considered as mesenchymal progenitor cells, capable of differentiation into fibroblasts or myofibroblasts, the main producers of collagen and elastin, and thus make contribution to the fibrotic lesions during wound repair [18, 12]. In agreement with previous reports, the current results documented elevated CD45+Col-1+ fibrocyte counts among patients with pulmonary fibrotic processes. In this series, we examined, for the first time, a greater than 5-fold increase in overall median fibrocyte counts among patients with BPD compared to normal subjects. The circulating fibrocytes in patients with ARDS did not show elevated, suggesting that the patients with ARDS would not develop fibrotic disease. In human subjects with interstitial pneumonia and IPF, elevated circulating fibrocytes in peripheral blood were detected. Furthermore, the number of circulated fibrocytes are associated with the severity of the disease [6, 15]. In accordance with this, the present study for the first time indicated increased fibrocytes in patients with BPD and shown a correlation between the number of fibrocytes and degree of hypoxia as well as degree of pulmonary hypertension (PH). The mechanism evidence support T-cell activation, possibly mediated by fibrocytes, are involved in this process [19].

For fibrocytes recruitment, the chemokine receptors CXCR4 and CCR7 appear to be pivotal for the homing of fibrocytes into the injured tissue [20, 21]. Elevated levels of CXCR4 and CCR7 in respiratory epithelial lining fluid has been reported in patients with obliterative bronchiolitis (OB) after lung transplantation (LTP) [22]. Increased immunoreactivity for CXCL12 in endothelial cells, macrophages, and T cells were detected in the airways of patients with asthma [23]. In a mouse model of bleomycin-induced lung injury, circulating CXCR4+ fibrocytes infiltrated and contributed to lung fibrosis after homing in response to the lung-plasma CXCL12 gradient [24], suggesting an active traffic through this pathway. We confirmed that a higher proportion of fibrocytes, nearly 80%, expressed CXCR4 in patients with BPD. The presence of these chemokine receptors further implied the essence of fibrocytes isolated with CD45 and Col-I positive from peripheral blood leukocytes. To our knowledge, there are no other studies demonstrating the presence of fibrocytes in the patients with BPD. At the same time, the CXCR4 receptors was presented at a high level in serum from patients with BPD. In previously clinical research, a major population of CXCR4+ human fibrocytes has been identified [25]. Moreover, neutralizing anti-CXCL12 antibodies resulted in reduced recruitment of circulating fibrocytes to the injured lung and attenuated fibrosis [26]. Our results revealed that cells that express fibrocyte markers are present in the peripheral blood of patients with BPD, potentially representing a process of migration and homing to the lung of these cell populations. A similar pathway seems to be followed in the recruitment of fibrocytes in IPF, similar to our report.

TGF-β1 is known to enhance the differentiation of blood derived pluripotential CD45+CD34+Col-I+ fibrocytes into SMA-expressing myofibroblasts [27]. Our data indicate that there were increased levels of TGF-β1 in the serum of patients with BPD compared with patients with ARDS and normal subjects, supporting the biological function of TGF-β1 in the development of fibrosis, which are similar to a report [28] showing that higher levels of TGF-β1 are produced by burn patient fibrocytes. In addition, our data indicate that TGF-β1 was elevated in BAL fluid, supporting this specific response to TGF-β1 for fibrocytes from patients with BPD. The media from burn patient fibrocytes could promote dermal fibroblast proliferation, migration, whereas a TGF-β1 neutralizing antibody might reduce this activity [29], supporting the concept that circulating fibrocyte could produce TGF-β1 and prime progenitor cells to proliferate and differentiate into SMA-expressing myofibroblasts.

Certain weaknesses should be considered in our study. First, due to our study design and the small sample size, it is difficult to draw a line between BPD and increased numbers of circulating fibrocytes (CD34+CD45+Col-I+) in the peripheral blood of patients. These data are preliminary and need to be validated by the presence of these cells in the lung parenchyma of the patients with BPD. Furthermore, at the time of initial admission for BPD, some of these patients had pre-existing BPD, whereas some patients are referred relatively early for BPD, so there exist wide variation for the timing of blood draws. Although these questions are still open, the current study has confirmed that circulating fibrocytes are present in the blood of patients with BPD, which grant their potential as biomarkers, regardless of their biological properties in detail.

In conclusion, we have found increased counts of fibrocytes and α-SMA positive myofibroblasts in the peripheral blood of patients with BPD compared with healthy control subjects and are associated with several clinical parameters. This study suggests a potential role for assaying fibrocytes as a biomarker for BPD evaluation; however, these findings should be confirmed or refuted by larger studies. Anymore, other studies are needed to understand the mechanisms behind the performance of fibrocytes in BPD lung fibrogenesis. The complementary studies on additive effects of fibrocytes and resident fibroblast on this process should be investigated in detail.
